# Recommendations for the management of MPS IVA: systematic evidence- and consensus-based guidance

**DOI:** 10.1186/s13023-019-1074-9

**Published:** 2019-06-13

**Authors:** Mehmet Umut Akyol, Tord D. Alden, Hernan Amartino, Jane Ashworth, Kumar Belani, Kenneth I. Berger, Andrea Borgo, Elizabeth Braunlin, Yoshikatsu Eto, Jeffrey I. Gold, Andrea Jester, Simon A. Jones, Cengiz Karsli, William Mackenzie, Diane Ruschel Marinho, Andrew McFadyen, Jim McGill, John J. Mitchell, Joseph Muenzer, Torayuki Okuyama, Paul J. Orchard, Bob Stevens, Sophie Thomas, Robert Walker, Robert Wynn, Roberto Giugliani, Paul Harmatz, Christian Hendriksz, Maurizio Scarpa, MPS Consensus Programme Steering Committee, Mehmet Umut Akyol, Tord D. Alden, Hernan Amartino, Jane Ashworth, Kumar Belani, Kenneth I. Berger, Andrea Borgo, Elizabeth Braunlin, Yoshikatsu Eto, Jeffrey I. Gold, Andrea Jester, Simon A. Jones, Cengiz Karsli, William Mackenzie, Diane Ruschel Marinho, Andrew McFadyen, Jim McGill, John J. Mitchell, Joseph Muenzer, Torayuki Okuyama, Paul J. Orchard, Bob Stevens, Sophie Thomas, Robert Walker, Robert Wynn, MPS Consensus Programme Co-Chairs, Roberto Giugliani, Paul Harmatz, Christian Hendriksz, Maurizio Scarpa

**Affiliations:** 1 Department of Otolaryngology, Hacettepe University, Ankara, Turkey; 2 Department of Neurosurgery, Ann & Robert H. Lurie Children’s Hospital of Chicago, Northwestern University Feinberg School of Medicine, Chicago, IL USA; 3 Child Neurology Department, Hospital Universitario Austral, Buenos Aires, Argentina; 4 Department of Paediatric Ophthalmology, Manchester Royal Eye Hospital, Manchester University NHS Foundation Trust, Manchester, UK; 5 Department of Anesthesiology, University of Minnesota, Minneapolis, MN USA; 6 Departments of Medicine and Neuroscience and Physiology, New York University School of Medicine, André Cournand Pulmonary Physiology Laboratory, Bellevue Hospital, New York, NY USA; 7 Orthopaedics Clinic, Padova University Hospital, Padova, Italy; 8 Division of Pediatric Cardiology, University of Minnesota, Minneapolis, MN USA; 9 Advanced Clinical Research Centre, Institute of Neurological Disorders, Kanagawa, Japan and Department of Paediatrics/Gene Therapy, Tokyo Jikei University School of Medicine, Tokyo, Japan; 10 Keck School of Medicine, Departments of Anesthesiology, Pediatrics, and Psychiatry & Behavioural Sciences, Children’s Hospital Los Angeles, Department of Anesthesiology Critical Care Medicine, 4650 Sunset Boulevard, Los Angeles, CA USA; 11 Hand and Upper Limb Service, Department of Plastic Surgery, Birmingham Women’s and Children’s Hospital, Birmingham, UK; 12 Willink Biochemical Genetic Unit, Manchester Centre for Genomic Medicine, St Mary’s Hospital, Manchester University NHS Foundation Trust, Manchester, UK; 13 Department of Anesthesiology and Pain Medicine, The Hospital for Sick Children, Toronto, Canada; 14 Department of Orthopedics, Nemours/Alfred I, Dupont Hospital for Children, Wilmington, DE USA; 15 Department of Ophthalmology, UFRGS, and Ophthalmology Service, HCPA, Porto Alegre, Brazil; 16 The Isaac Foundation, Campbellford, Ontario Canada; 17 Department of Metabolic Medicine, Queensland Children’s Hospital, Brisbane, Australia; 18 Division of Pediatric Endocrinology, Montreal Children’s Hospital, Montreal, QC Canada; 19 Department of Pediatrics, School of Medicine, University of North Carolina at Chapel Hill, Chapel Hill, NC USA; 20 Department of Clinical Laboratory Medicine, National Centre for Child Health and Development, Tokyo, Japan; 21 Division of Blood and Marrow Transplantation, Department of Pediatrics, University of Minnesota, Minneapolis, MN USA; 22 MPS Society, Amersham, Buckinghamshire UK; 23 Department of Paediatric Anaesthesia, Royal Manchester Children’s Hospital, Manchester, UK; 24 Department of Paediatric Haematology, Royal Manchester Children’s Hospital, Manchester, UK; 25 Department of Genetics, UFRGS, and Medical Genetics Service, HCPA, Porto Alegre, Brazil; 26 UCSF Benioff Children’s Hospital Oakland, Oakland, CA USA; 27 Steve Biko Academic Hospital, University of Pretoria, Pretoria, South Africa; 28 Center for Rare Diseases at Host Schmidt Kliniken, Wiesbaden, Germany and Department of Paediatrics University of Padova, Padova, Italy

**Keywords:** Morquio a syndrome, Mucopolysaccharidosis, MPS IVA, Management guidelines, Elosulfase alfa, VIMIZIM, Enzyme replacement therapy, ERT, Haematopoietic stem cell transplantation, HSCT, Surgery, Anaesthetics

## Abstract

**Introduction:**

Mucopolysaccharidosis (MPS) IVA or Morquio A syndrome is an autosomal recessive lysosomal storage disorder (LSD) caused by deficiency of the *N*-acetylgalactosamine-6-sulfatase (GALNS) enzyme, which impairs lysosomal degradation of keratan sulphate and chondroitin-6-sulphate. The multiple clinical manifestations of MPS IVA present numerous challenges for management and necessitate the need for individualised treatment. Although treatment guidelines are available, the methodology used to develop this guidance has come under increased scrutiny. This programme was conducted to provide evidence-based, expert-agreed recommendations to optimise management of MPS IVA.

**Methods:**

Twenty six international healthcare professionals across multiple disciplines, with expertise in managing MPS IVA, and three patient advocates formed the Steering Committee (SC) and contributed to the development of this guidance. Representatives from six Patient Advocacy Groups (PAGs) were interviewed to gain insights on patient perspectives. A modified-Delphi methodology was used to demonstrate consensus among a wider group of healthcare professionals with experience managing patients with MPS IVA and the manuscript was evaluated against the validated Appraisal of Guidelines for Research and Evaluation (AGREE II) instrument by three independent reviewers.

**Results:**

A total of 87 guidance statements were developed covering five domains: (1) general management principles; (2) recommended routine monitoring and assessments; (3) disease-modifying interventions (enzyme replacement therapy [ERT] and haematopoietic stem cell transplantation [HSCT]); (4) interventions to support respiratory and sleep disorders; (5) anaesthetics and surgical interventions (including spinal, limb, ophthalmic, cardio-thoracic and ear-nose-throat [ENT] surgeries). Consensus was reached on all statements after two rounds of voting. The overall guideline AGREE II assessment score obtained for the development of the guidance was 5.3/7 (where 1 represents the lowest quality and 7 represents the highest quality of guidance).

**Conclusion:**

This manuscript provides evidence- and consensus-based recommendations for the management of patients with MPS IVA and is for use by healthcare professionals that manage the holistic care of patients with the intention to improve clinical- and patient-reported outcomes and enhance patient quality of life. It is recognised that the guidance provided represents a point in time and further research is required to address current knowledge and evidence gaps.

**Electronic supplementary material:**

The online version of this article (10.1186/s13023-019-1074-9) contains supplementary material, which is available to authorized users.

## Background

Mucopolysaccharidoses (MPS) are a clinically heterogeneous group of rare metabolic disorders referred to as lysosomal storage disorders (LSDs). MPS disorders arise due to deficiencies in enzymes that break down glycosaminoglycans (GAGs) [1–3]. The build-up of GAGs, either directly or indirectly, cause progressive damage to cells, tissues and various organ systems and result in severe morbidity and reduced life expectancy [4, 5]. Clinical features that are common to all MPS disorders include skeletal and joint abnormalities, dysfunction in vision and hearing, cardiorespiratory problems, hepatosplenomegaly and coarse features [1]. Changes in the central nervous system are characteristic of some of these disorders, with typical imaging findings including white matter lesions, hydrocephalus, cervical spinal canal stenosis and bone abnormalities of the skull and spine [6]. MPS IVA or Morquio A syndrome (253000) is an autosomal recessive MPS disorder caused by deficiency of the *N*-acetylgalactosamine-6-sulfatase (GALNS) enzyme (EC 3.1.6.4), which impairs lysosomal degradation of keratan sulphate and chondroitin-6-sulphate [7–11]. Results of a systematic review indicate that the point prevalence of MPS IVA was 1 per 926,000 in Australia, 1 per 1,872,000 in Malaysia and 1 per 599,000 in the UK [12]. Many genetic mutations have been identified and are responsible for the heterogeneous clinical presentation, which make diagnosis challenging. Although elevated urinary GAGs or reduced GALNS activity in dried blood spot results may be indicative of MPS IVA, a definite diagnosis is often only achieved through demonstration of reduced GALNS activity in leukocytes or fibroblasts or with molecular confirmation of two disease-causing mutations in the *GALNS* gene [13].

MPS IVA is a heterogeneous and progressive disorder. Skeletal and joint abnormalities including genu valgum, joint hypermobility, hip subluxation and dysplasia, spinal cord compression, spinal instability and thoracolumbar kyphoscoliosis are the most prevalent manifestations [8, 14–16], and patients require regular assessment of multi-organ involvement [17]. Respiratory impairment and spinal cord instability are the main cause of morbidity and mortality [14, 18, 19] and cardiac dysfunction has also been reported [20]. Other manifestations include corneal clouding and hearing loss.

## Objectives

This manuscript provides robust guidance for the management of adult and paediatric patients with MPS IVA using a validated modified-Delphi methodology to gain consensus. The guidance is comprised of a holistic set of recommendations for the timely and appropriate use of medical and surgical interventions and management of the natural history of MPS, with the intention to maintain and enhance patient quality of life with the intention to maintain and enhance patient quality of life (QoL) and improve and improve clinical- and patient reported- outcomes. The guidance is intended for use by healthcare professionals that manage the care of patients with MPS IVA, in particular paediatricians and geneticists, and aims to enhance multidisciplinary practice across specialities. It also provides specific guidance for other specialists (Table 1) and stakeholders in the health services who are in contact with patients with MPS and is a useful reference for patient advocates, patients and their families. Table 1 describes the areas of clinical focus covered within this guidance and the corresponding recommended speciality focus.

**Table 1:** Clinical areas of focus and recommended speciality focus

Table	Recommended speciality focus
Table 2: General principles for the management of patients with MPS IVA	all
Table 3: Recommended routine monitoring and assessments in patients with MPS IVA	all
Table 4: Guidance statement for elosulfase alfa	geneticist, metabolic physician, paediatrician, nurse, physiotherapist
Table 5: Guidance statement for HSCT	anaesthetist, bone marrow transplant expert/hematopoietic stem cell transplant expert, geneticist, paediatrician, nurse
Table 6: Guidance statements for CPAP, NIPPV, oxygen supplementation and hypercapnia monitoring	anaesthetist, ear-nose-throat specialist, geneticist, paediatrician, respiratory physician/pulmonologist, nurse
Table 7: Guidance statements for anaesthesia	all
Table 8: Guidance statements for hip reconstruction, hip replacement and growth modulation surgeries	anaesthetist, geneticist, orthopaedic surgeon, neurosurgeon, paediatrician, physiotherapist
Table 9: Guidance statements for decompression of the spinal cord, spinal stabilisation and thoracolumbar kyphoscoliosis	anaesthetist, geneticist, orthopaedic surgeon, neurosurgeon, paediatrician, physiotherapist
Table 10: Guidance statement for corneal transplantation	anaesthetist, geneticist, ophthalmologist, paediatrician
Table 11: Guidance statement for cardiac valve replacement	anaesthetist, cardiologist, geneticist, paediatrician
Table 12: Guidance statements for tonsillectomy and/or adenoidectomy, tracheostomy and insertion of ventilation tubes	anaesthetist, geneticist, ear-nose-throat specialist, paediatrician, respiratory physician/pulmonologist

This guidance was developed as part of a broader consensus programme that also covered the management of MPS VI, the results of which are published in a companion article (*Recommendations for the management of MPS VI: systematic evidence- and consensus-based guidance*).

## Methods and process

The methods described below cover both MPS IVA and MPS VI; however, the results section focuses on MPS IVA only.

### Convening of the steering committee

Four steering committee (SC) Co-Chairs were appointed using a systematic expert mapping approach based on a ranking measure of publication/congress activity relevant to the management of MPS IVA/VI and leadership roles in editorial boards and professional bodies. The Co-Chairs made recommendations for 22 SC members from across the world, including experts in: anaesthesia, ear nose and throat (ENT) surgery, cardiology, genetics, endocrinology, hand surgery, HSCT, neurosurgery, ophthalmology, orthopaedic surgery, paediatrics, pain management and pulmonology. To ensure the patient view was represented, three members from Patient Advocacy Groups (PAGs) formed part of the SC group. The SC defined the scope of the programme, including the medical and surgical interventions to be covered in the guidance and provided search terms for the literature review. Further details, including the competing interests, institutions and contributions of each SC member are listed within the declarations section of this manuscript.

### Setting the clinical questions to be addressed by the guidance

The clinical questions to be addressed by the guidance were developed by the SC group according to the patient, interventions, comparator and outcome (P.I.C.O.) methodology (Additional file 1: Appendix 1a) and are shown below.

1. What are the general principles for the management of adult and paediatric patients with MPS IVA/VI?
2. What are the recommended routine monitoring and assessments that should be used to track the natural history of adult and paediatric patients with MPS IVA/VI and indicated interventions to be used during the care of the common symptoms of MPS?
3. For adult and paediatric patients with MPS IVA/VI, what is the impact on clinical outcomes and safety/tolerability of:

- Interventions that address the underlying enzyme deficiency including:
  - ERT
  - HSCT
- Interventions used to manage the symptoms of MPS IVA/VI including:
  - Respiratory and sleep disorders
  - Anaesthesia
  - Limb and spinal surgeries
  - Ophthalmic surgeries
  - Cardio-thoracic surgeries
  - ENT surgeries

A secondary focus of the programme was to highlight current evidence gaps and provide recommendations for future treatment directions. Topics that were deemed out of scope of this programme included: comprehensive recommendations for diagnosis (e.g. screening, testing and identification of differential disease phenotypes), validation of new clinical outcome assessment tools (e.g. to assess patient-reported outcomes) and defining minimal clinically important differences for MPS IVA/VI.

### Patient Advocacy Groups insights

Consultations were conducted with representatives from six global PAGs (including Brazil, Canada, Germany, Turkey, the United Kingdom and the United States) [listed in the acknowledgements section of the manuscript]. To gain an understanding of the experience and views of patients with MPS IVA/VI on the holistic management of MPS across several geographies and healthcare systems, the consultations addressed: the biggest challenges faced by patients with MPS IVA/VI; the impact of medical/surgical interventions on patient outcomes, including: physical function/performance, mental/emotional outcomes and socioeconomic outcomes/cost implications; the overall benefit/risk profile associated with medical/surgical interventions and how this influences patient choice; barriers to the use of medical/surgical interventions across each region (e.g. reimbursement challenges, cost issues, access issues etc) and facilitators and barriers for application of the guidance.

### Systematic literature review methodology

A systematic literature review was independently conducted by three Biographical Fellows in accordance with the Preferred Reporting Items for Systematic Reviews and Meta-Analyses (PRISMA) statement [21]. The focus of the literature review was to collate evidence for the clinical questions. Owing to the nature of the recommendations for general principles and routine monitoring and assessments, the associated guidance is predominantly based on clinical opinion; however, where available, published evidence has been applied to support these statements.

Literature searches were performed in July 2017 using PubMed, Web of Science, SCOPUS and the Cochrane Database of Systematic Reviews; no early date limit was set. Supplementary searches of Google Scholar were undertaken to identify recent publications. Search strings incorporated Medical Subject Headings and free text key words, and were defined based on the P.I.C.O. methodology to answer each of the clinical questions (Additional file 1: Appendix 1a) [22]. Reproducible search strategies were reviewed by the SC (Additional file 1: Appendix 1b)**.** As part of the screening, duplicates were removed. Exclusion criteria included: in-vitro/animal studies, non-English language studies, studies that did not report an outcome, diagnosis studies, non-systematic review articles and studies that did not relate to a specific intervention. Only studies conducted in humans were included and evidence was extrapolated from other MPS types. Single case studies were captured and referred to where evidence was scarce. The bibliographies of identified pre-existing guidelines and review articles were checked for additional relevant studies. The following information was extracted from included studies: reference details, patient population, intervention, comparator (where applicable), outcomes (physical function/performance, mental/emotional, socioeconomic), safety and tolerability and reported study limitations. Data were extracted by one Bibliographic Fellow and checked by a second. Any discrepancies in screening and data extraction were resolved through discussion or the intervention of a third reviewer. Results were reported according to PRISMA (Additional file 1: Appendices 1b and 1c).

The quality of evidence level for each paper was assessed using the Oxford Centre for Evidence-based Medicine (OCEM) criteria. In accordance with the criteria (Additional file 1: Appendix 2) each reference obtained from the systematic literature review was critically appraised and designated an evidence level based on the study design, rigour of methodology and outcomes (Additional files 2 and 3). The recommendation statements were then graded based on the average evidence level for each supporting reference (Additional files 2 and 3). The evidence levels and grades were cross-checked by the SC group and used to assess the strength of each recommendation during the development of the guidance statements.

### Development and validation of guidance statements

For each clinical area of focus and intervention, the SC developed draft guidance statements accompanied by supporting text, which were based on the results of the literature search, insights from the PAG interviews and expert clinical opinion. This process was facilitated by an independent secretariat via a series of face-to-face and online meetings and email correspondence across a 14-month period.

Guidance statements were ratified using a modified-Delphi voting process via an online survey [23, 24]. To ensure specialists were engaged from a wide range of geographies and across numerous medical and surgical specialities, the survey containing all guidance statements was sent by the independent secretariat to 197 MPS physicians across 35 clinical areas of focus in 24 countries. Three reminders were sent requesting survey participation across a three-week open voting period. Participation in the survey was voluntary and anonymous to eliminate bias (a summary of respondents by specialities and country can be found within Additional files 4 and 5). The names of respondents were not collected; however, respondents were required to provide information pertaining to their hospital/institution, speciality, country, years’ experience in the management of MPS patients and number of MPS IVA and MPS VI patients they manage/have managed. Respondents were required to meet a minimum threshold of experience in the management of patients with MPS, which was determined by the SC prior to the initiation of the survey to be: (1) for metabolic specialists: at least 5 years’ experience of managing numerous patients with MPS (preferably MPS IVA/VI); (2) for specialist surgeons/anaesthetists: at least 3 years’ experience of managing numerous patients with MPS (preferably MPS IVA/VI). Respondents were asked to vote on statements within their area of expertise only and were able to opt-out of statements outside of their scope of medical practice speciality. Guidance statements were voted on using a Likert scale from 1 (strongly disagree) to 9 (strongly agree). Scores of 6 or less were defined as disagreement with the statement and it was mandatory for respondents to provide rationale for their disagreement and suggest amendments to the statement. Scores of 7 or more were defined as agreement with the statement and respondents were given the option to add comments.

Consensus was reached when ≥75% of respondents agreed with a given statement. This consensus threshold was determined by review of literature and applied to the rare disease field by the Steering Committee group. The most commonly reported definition of consensus for Delphi studies is percent agreement, with 75% being the median threshold to define consensus [24].

Statements for which consensus was not achieved in the first round of voting were amended by the SC based on respondent feedback and expert clinical opinion and sent for re-voting. The percentage consensus and assigned evidence grade for each guidance statement is shown within the results section below.

The quality of study reporting in this programme was independently assessed by three reviewers using the Appraisal of Guidelines for Research and Evaluation (AGREE) II Instrument (declared in the acknowledgements section of the manuscript) [25].

### Measures to ensure independence

The programme was funded by BioMarin; the manufacturer of the recombinant enzymes used to treat MPS IVA and MPS VI, however, they were not involved in any stages of the process and did not influence the scope or content of the programme. BioMarin were absent from all SC meetings, were blinded to the guidance statements and were not involved in the publication process. The programme was managed by an independent secretariat (Lucid Partners Ltd), and the scope of the programme and content, including the development of guidance statements, was led by the SC with editorial support provided by the secretariat. The SC was identified through a systematic expert mapping process, conducted independently of the funder. Conflicts of interests for all SC members (shown in the declarations section) were recorded at the start of the consensus programme and updated throughout the programme. Following the systematic expert mapping exercise, it was noted that some of the SC had previously worked on a consultancy basis with the programme sponsor, who hold the marketing authorisation for approved pharmaceutical therapy in MPS IVA. Efforts were therefore taken to ensure representation from leading experts across other treatment modalities, including HSCT/BMT on the Steering Committee panel and during the modified-Delphi voting process, where a large number of physicians across multiple specialities and geographies were engaged. At several stages during the process, the SC were also required to provide updated conflict of interest disclosures.

## Results

Here we report the results relevant to the management of patients with MPS IVA. The results for MPS VI are published in a companion article (*Recommendations for the management of MPS VI: systematic evidence- and consensus-based guidance*).

### Patient Advocacy Groups insights

The results of the PAG consultations highlighted that the biggest current unmet needs and challenges faced for patients with MPS IVA/VI include: treatment expertise, timing and access to treatment, appropriate infrastructure, maintenance of independence and social prejudice. The results also summarised the factors that affect patient prognosis and QoL, key considerations for all surgical interventions with an emphasis of anaesthesia, barriers to use of medical/surgical interventions and patient and caregiver considerations.

Detailed insights from all PAG consultations were presented to the SC group by the patient advocates who formed part of the SC and were used to inform the development of the guidance statements and ensure representation of the patient voice. These insights assisted with the development of the general principles for management of patients with MPS IVA/VI, and ensured holistic care was considered across the whole guidance development process.

### Modified-Delphi results

The modified-Delphi survey contained 116 guidance statements covering both MPS IVA and VI; only the MPS IVA statements are described below (Tables 2, 3, 4, 5, 6, 7, 8, 9, 10, 11, 12). In round one of the modified-Delphi voting, the online survey was sent to 197 MPS physicians (nominated by the SC group), including all members of the SC. A total of 103 submissions were received from 82 institutions across 20 countries (further information including the number of respondents/statements, respondent specialities and geographies, and respondent feedback to guidance statements are included within Additional file 4). Seven respondents did not meet the minimum threshold of experience and their submissions were excluded. According to the consensus criteria threshold of 75%, 103/116 statements reached consensus (Additional file 4). The guidance statements that did not reach consensus were reviewed by the SC and amended based on feedback from the modified-Delphi participants and expert clinical opinion.

In round two of the modified-Delphi voting, 13 statements were sent for re-voting to 145 MPS physicians (experts of specialties not relevant to these statements were removed from the mailing list). A total of 71 survey submissions were received from 53 institutions across 18 countries and two respondents did not meet the minimum experience threshold and their submissions were excluded (further information including the number of respondents/statements, respondent specialities and geographies, and respondent feedback to guidance statements are including within Additional file 5). Consensus was reached on all 13 statements (Additional file 5). In total, 87 guidance statements reached consensus for MPS IVA.

### Appraisal of Guidelines for Research and Evaluation (AGREE) II assessment

The methodological rigour and transparency employed within the development of this guidance was evaluated through the review of the manuscripts against the validated AGREE II instrument. Three independent reviewers [listed in the acknowledgements section of the manuscript] assessed the manuscript and suggested amendments were addressed where possible; a subsequent second round of review was conducted by all reviewers. Across each AGREE II criteria, the average domain scores obtained were: scope and purpose (85%), stakeholder involvement (85%), rigour of development (73%), clarity of presentation (78%), applicability (34%) and editorial independence (64%) (full information including the scores from the two rounds of AGREE II evaluation can be found in Additional file 1: Appendix 3). The guidance documents were given an overall guideline assessment score of 5.3/7 (where 1 represents the lowest quality, and 7 represents the highest quality).

### Guidance statements

#### General principles (Table 2)

The SC noted that newborn screening in MPS IVA would allow earlier diagnosis if/when available thus leading to earlier intervention, which would likely change the course of disease. As comprehensive recommendations for diagnosis of MPS IVA is out of scope of this guidance, more detailed discussions of newborn screening can be found elsewhere [17]. General guidelines for pain management in patients with MPS have also been published elsewhere [26, 27].

**Table 2:** General principles for the management of patients with MPS IVA

Statement	Percentage consensus
All guidance statements are evidence Grade D (level 5 expert clinical opinion)	
Diagnosis of MPS IVA during infancy is critical to optimise patient outcomes	98%
The first consultation should be conducted by a physician with experience of treating MPS as soon as possible after diagnosis. This should include a full discussion of disease pathology, progression, treatment options and management. Ongoing information should be provided to optimise patient outcomes	97%
Patients and caregivers should receive ongoing psychosocial support from a social worker and/or psychologist, and should be directed towards an MPS society or relevant patient organisation in their country	94%
A comprehensive medical history and multi-system evaluation should be conducted within days of diagnosis to set a baseline for ongoing assessments and evaluate the physical and neurological manifestations of disease, functional ability and disease burden	88%
Ongoing and regular multi-system monitoring, and assessments are recommended to track the natural history of MPS IVA, monitor the impact of treatment and assess the need for treatment interventions to manage the symptoms of MPS IVA. These should be conducted at every clinic visit, annually or in some cases as clinically indicated (for example pre- and post-operatively)	100%
Timely interventions are recommended where clinically indicated by monitoring, to help avoid irreversible damage caused by the natural history of MPS IVA, and to manage the disease manifestations and maintain long-term QoL	99%
A multidisciplinary team (MDT) of metabolic specialists, surgeons and allied healthcare professionals (including, but not limited to: nurses, physiotherapists, occupational therapists, psychologists and audiologists) is required to manage the diverse range of disease manifestations of MPS IVA	99%
Co-ordination of the entire MDT care team is required prior to any procedure to determine the need for surgery, to discuss the benefits and risks of combining surgeries to minimise the need for multiple anaesthetics and to decide the optimal order of procedures. The decision to combine surgeries should take into consideration the surgical and intubation time, and complexity of procedures	93%
The risks and benefits of any intervention and the competing risks of other medical problems should be assessed and discussed with patients, families and caregivers such that they can make an informed decision on the appropriateness of the therapy/surgery	100%
Surgical procedures should be performed by (or under the guidance of) specialist surgeons and anaesthetists with experience of MPS, in medical centres with intensive care units	99%
Management of pain should be a fundamental part of the care of patients with MPS IVA, with the aim of improving QoL and maintaining mobility. Refer to general guidelines for pain management	100%

#### Recommended routine monitoring and assessments (Table 3)

**Table 3:** Recommended routine monitoring and assessments in patients with MPS IVA

Statement	Percentage consensus
All guidance statements are evidence Grade D (level 5 expert clinical opinion), unless otherwise stated	
Physical examination	
A physical examination should be performed during every visit to assess general health, growth, vital signs, abdominal organ size, presence of hernia, neurologic function (including gait), ligamentous laxity, and functions of the eyes, ears, heart and lungs	90%
Routine physical examination can also identify signs of potential respiratory problems, such as an enlarged tongue or sniffing position	90%
Radiology	
While X-rays are essential to identify the natural history of disease and response to treatment, efforts should be made to minimise radiation exposure, and images should be requested only when clinically useful	85%
Hips: an anteroposterior (AP) pelvis radiograph should be performed at diagnosis and as clinically indicated (based on physical examination or reports of pain) to quantify hip dysplasia or identify early signs of hip migration	88%
Lower limbs: in patients with clinical evidence of valgus deformity of the lower limbs, standing AP radiographs of lower extremities should be performed prior to guided growth surgery	100%
Spine: standing or sitting plain radiography of the cervical and thoracolumbar spine to examine for spinal deformities is recommended in patients with MPS IVA at diagnosis and every 2–3 years thereafter, or sooner if clinically indicated	85%
Magnetic resonance imaging (MRI) of the whole spine (in neutral position) should be performed annually in children with MPS IVA to assess for spinal cord injury. The frequency may be reduced for adult patients with stable imaging who do not display symptoms^a^	84%
Flexion/extension MRI of cervical spine may be needed to identify changes in spinal canal and spinal cord	86%
MRI of the brain is recommended at diagnosis in patients with MPS IVA, and should be repeated as needed in individuals with clinical suspicion of hydrocephalus	80%
MRI of the brain and spinal cord in patients with MPS IVA may require sedation or general anaesthesia, depending on patient age and cooperation. General anaesthesia carries substantial risk for patients with MPS	95%
Flexion/extension computerised tomography (CT) of the craniocervical junction may be considered in patients with MPS IVA if MRI is not available or if sedation is not possible	92%
The presence of specific radiological signs may indicate the need for surgical intervention to correct skeletal deformities; however, there is insufficient evidence to support preventative surgery based on radiological findings	88%
Endurance	
Choice of assessment depends on the patient’s physical and developmental ability	97%
Baseline assessment is the most important and ideally two values should be obtained as a minimum. Consistent protocols should be used when performing repeat measurements to minimise variability	95%
Annual endurance testing using the 6-min walk test (6MWT) is recommended, as per the American Thoracic Society guidelines [1, 45] Evidence Grade: C (level 4 study and extrapolation from level 1 study) [8, 46]	87%
In patients with limited ambulation who are unable to perform the 6MWT, endurance should be assessed via alternative methods such as an adapted timed 25-ft walk test (T25FW)	76%
Endurance testing is also recommended prior to initiation of ERT and annually thereafter as a measure of treatment efficacy and to provide early evidence of possible neurologic or skeletal issues	87%
Growth	
Assessment of growth should be performed at each clinic visit (ideally every 6 months) as part of a regular physical examination and should include: standing height (sitting height if the patient is unable to stand), length (supine position), weight, head circumference (≤3 years), Tanner pubertal stage (until maturity) [47]	95%
Height and weight should also be measured before initiation of ERT and at every clinic visit thereafter (ideally every 6 months) to evaluate the impact of treatment [47]	95%
Urinary keratan sulphate (KS)/urinary glycosaminoglycan (uGAG) levels	
Where available, tandem mass spectrometry may be used to assess levels of urinary keratan sulphate prior to starting elosulfase alfa and every 6 months thereafter to determine the pharmacodynamic effects of ERT [48] Evidence Grade: D (level 3/4 studies support the statement; [8, 49–54] however, one level 3 study [55] does not support use of urinary keratan sulphate for monitoring the therapeutic effect of ERT)	94%
Total uGAG levels are often elevated in neonates and infants with MPS IVA and may overlap with normal values in adults and some teenagers. However, if a specific keratan sulphate assay is not available, measurement of uGAG levels using standard dye-binding methods may be useful. Preferably, measurements should be performed in the same laboratory and assessed against age-related reference values	85%
Cardiac function	
Initial cardiac evaluation should be performed at the time of diagnosis and include assessment of vital signs with measurement of oxygen saturation, right arm and leg blood pressure measurements, careful auscultation, full transthoracic two-dimensional and Doppler echocardiogram, and 12-lead electrocardiogram (ECG)	100%
Longer ECG monitoring (prolonged Holter/event monitoring) may be considered in older patients, especially if they have symptoms of black outs, unexpected falls or dizziness	96%
Follow-up in expert centres should be annually initially, but may be extended to every 2–3 years if there is no evidence of cardiac abnormality	92%
Additional cardiac assessment, including a standard ECG^b^, should be performed prior to any surgical procedure requiring general anaesthesia	92%
Neurological exam	
A detailed neurological examination should be performed at every clinic visit (minimally every 6 months) and, where possible, these should correlate with imaging studies of the spine to detect early spinal stenosis or instability compromising the cervical cord. For patients without clinical or radiographic concern, annual neurological examination may be sufficient [56]	87%
Standard MRI of the cervical spine should be performed to assess for presence of spinal cord compression. In the absence of significant spinal cord compression, proceed with flexion/extension MRI to confirm the presence of worsening spinal cord compression with motion^c^	78%
Respiratory function and sleep disorder	
Evaluation of respiratory function by spirometry, including forced vital capacity (FVC) and maximum voluntary ventilation (MVV), should be performed to assess changes in lung volume and obstruction in children over 5 years of age	97%
Respiratory function should be assessed annually until children stop growing, and every 2–3 years thereafter, provided that respiratory symptoms remain unchanged. Additional testing should be performed if respiratory symptoms change or if intercurrent illnesses occur	91%
Normative values are not available, therefore change in absolute volume from patient’s own baseline will be the best indicator of deterioration or improvement	97%
Measurement of respiratory rate and arterial oxygen saturation before and after annual endurance testing is recommended	86%
Evaluation of gas exchange and respiratory function is also recommended before any planned air travel, to ensure safety during the flight	86%
To identify symptoms of sleep apnoea^d^, patients should be asked to report presence of snoring and morning headaches at every clinic visit	100%
An overnight sleep study (polysomnography) is recommended at diagnosis (if possible, and no later than 2 years of age), and every 3 years thereafter or when signs and symptoms of obstructive sleep apnoea (OSA) are noted	94%
Ear-nose-throat (ENT)	
ENT examination, including tympanometry^e^, should be conducted every 3–6 months during childhood and every 6–12 months thereafter	91%
ENT examination in patients with MPS IVA should include visualisation of the upper respiratory tract to determine diagnosis, management and assist in pre-operative planning. Endoscopic examinations should be recorded and kept, to monitor disease progression	92%
Fibreoptic examination in patients with MPS IVA should be performed at diagnosis and at least annually thereafter, or as clinically indicated. For those individuals who require general anaesthesia, ENT examination should be performed during the pre-operative evaluation for other surgical procedures	83%
Upper airway CT, focused on airway anatomy preferably with reconstruction, may be useful to identify the area of the abnormality and possible cause of obstruction in patients with MPS IVA with suspected obstruction or malacia^f^	92%
Age-adjusted audiometric assessment as a baseline objective hearing evaluation should be conducted at first clinic visit and repeated annually to assess conductive and sensory-neural hearing loss Evidence Grade: C (Grade 4 studies) [57, 58]	100%
If speech problems are determined during ENT examination, an assessment by a speech pathologist should be conducted [59]	100%
Balance tests should be conducted if the patient has a history of balance problem	95%
Ophthalmological function	
Age-appropriate evaluations by an ophthalmologist is recommended every 6 months if possible, or at least annually [60]	90%
Ophthalmic assessment may include visual acuity, refraction, slit-lamp examination of cornea, funduscopic evaluation including optic nerve, and measurement of intraocular pressure	100%
Scotopic and photopic electroretinogram may be performed in patients with clinical suspicion of retinopathy or when considering corneal transplantation [60]	100%
Intraocular pressure monitoring and pachymetry may be considered prior to corneal transplant [60]	100%
Evaluation of oral health by dentist	
Close monitoring of dental development (at least annually) is recommended to prevent caries and attrition, as is monitoring of occlusion and chewing functions	100%
The need for subacute bacterial endocarditis (SBE) prophylaxis prior to dental procedures should be assessed by a cardiologist	100%
Disease burden	
Annual assessment of patient-reported outcomes is recommended for: pain severity, QoL (as assessed by reproducible and age-appropriate questionnaires [e.g. EQ-5D-5 L]), fatigue), and activities of daily living (ADL; as assessed by functional tests [6MWT/T25FW]), age-appropriate ADL questionnaires (e.g. MPS Health Assessment Questionnaire [MPS HAQ]), and assessment of wheelchair/walking aid use [61]	97%
These assessments may have to be adapted both for language, culture, and individual physical limitations, as they have not been validated in specific disorders	97%
Physical therapy	
Regular assessments by a physical therapist (lower limb), occupational therapist (upper limb) and rehabilitation medicine specialist should be conducted to assess limb function and provide support as needed	93%
The physical therapist could also assist in suggesting walking aids and other adaptations that may improve QoL	98%

Post-consensus comments by the SC to be taken into consideration:

^a^Magnetic resonance imaging (MRI) can also be used to assess for spinal cord compression. The frequency may be reduced for older patients with stable imaging who do not display symptoms

^b^Echocardiogram (ECHO) should also be performed prior to any surgical procedure requiring general anaesthesia

^c^This topic was discussed in detail with the neurosurgical and orthopaedic colleagues in the SC group. It was their expert clinical opinion that flexion/extension MRI is not dangerous to perform within the hands of an experienced team. It is important that the range of motion (ROM), flexion and extension of the patient is evaluated while they are awake immediately before anaesthesia. The ROM during anaesthesia should not exceed the ROM as noted in the awake state, and should only be carried out after it is confirmed that there is no spinal cord compression. See Table 9 for guidance statements on spinal surgeries including spinal cord decompression

^d^Signs and symptoms for sleep apnoea (a type of sleep disordered breathing (SDB)) can be divided into nocturnal and daytime symptoms. Nocturnal symptoms include loud snoring, observed episodes of breathing cessation during sleep, abrupt awakenings accompanied by gasping or choking, and awakening with a dry mouth or sore throat. Daytime symptoms include excessive daytime sleepiness, morning headaches, difficulty concentrating during the day, personality and mood changes including depression or irritability, and high blood pressure. To identify presence of SDB, patients should be asked to report snoring and other signs and symptoms of SDB at every clinic visit

^e^Tympanometry is used to measure the volume of the ear canal/tympanic membrane movement and indirectly assess for fluid accumulation and opening of pressure equalising tubes

^f^Upper airway CT may also be useful to identify the area of the abnormality and possible cause of obstruction in patients with MPS IVA with suspected tracheo bronchomalacia

#### Disease-modifying interventions

##### ERT (elosulfase alfa) in patients with MPS IVA (Table 4)

**Table 4:** Guidance statement for elosulfase alfa

Statement	Percentage consensus
Initiation of long-term ERT with elosulfase alfa at a dose of 2.0 mg/kg/week through intravenous infusion is recommended in all patients with MPS IVA as soon as possible after a confirmed diagnosis Evidence Grade: B (level 2 or 3 studies)	79%

***Rationale and evidence base*** Elosulfase alfa [28] is a recombinant form of the human lysosomal enzyme GALNS, which is deficient in patients with MPS IVA [29]. ERT with elosulfase alfa aims to transiently restore GALNS activity, thereby preventing the accumulation of KS and chondroitin-6-sulphate in lysosomal compartments of cells, which is responsible for the clinical manifestations of MPS IVA. Elosulfase alfa is currently the only disease-specific treatment that is licensed for patients with MPS IVA, having been validated in clinical trials [30–32]. In a Phase III clinical trial, patients who received intravenous elosulfase alfa at a dose of 2 mg/kg/week experienced reductions in urinary levels of keratan sulphate (a pharmacodynamic biomarker for the disease) [30]. Elosulfase alfa has been shown to improve endurance and exercise capacity (as measured by the 6MWT), which may in part relate to improved respiratory function and oxygen utilization [30, 33–36]. A trend for improvement in performance of ADL was also observed in a long-term extension study. The findings suggest that long-term elosulfase alpha ERT is associated with partial recovery of functional abilities, improving Morquio A patients’ abilities to perform ADL [36]. The evidence for the effect of elosulfase alfa on bone is currently limited and further research is required.

Elosulfase alfa is well-tolerated [37] and results of a randomized, double-blind, pilot study has shown that treatment reduces pain in some patients with MPS IVA [38]. The majority of adverse events in clinical trials were infusion associated reactions (IARs), which are defined as reactions occurring after initiation of infusion until the end of the day following the infusion. Serious IARs were observed in clinical trials and included anaphylaxis, hypersensitivity and vomiting [28]. As stated on the US Prescribing Information for elosulfase alfa, the most common symptoms of IARs (occurring in ≥10% of patients treated with elosulfase alfa and ≥ 5% more when compared with placebo) were headache, nausea, vomiting, pyrexia, chills and abdominal pain. IARs were generally mild or moderate and the frequency was higher during the first 12 weeks of treatment and tended to occur less frequently with time [28, 39].

Early intervention with elosulfase alfa is associated with a trend towards improvement in growth however the data is currently limited [40]. Most data related to the use of elosulfase alfa are from patients who initiated ERT relatively later in their disease. The early initiation of ERT will likely change the course of disease in patients with MPS IVA; therefore, additional studies are warranted to determine the long-term outcomes of patients in whom elosulfase alfa treatment is administered from an early age.

***Considerations prior to starting ERT*** It is important to evaluate the life-long impact of elosulfase alfa on an individual basis, as the benefits of treatment may not be consistent across all patients and there may be subpopulations of patients where the risk-benefit and efficacy versus cost effectiveness is less certain (e.g. less severe phenotypes) [41]. The status of the patient, disease burden, comorbidities and prognosis should be fully considered prior to initiation. The first dose of elosulfase alfa should (where possible) be administered by a clinician with experience of metabolic disorders and take place within an infusion centre/hospital with facilities for effective management of allergic/anaphylactic reactions. Home infusion may be considered where possible; this decision should be made by the physician and patient. Careful patient selection, good vascular access and a detailed management plan for IARs and anaphylaxis are essential for the success of this approach [42]. Consideration should be given to the need for a totally implantable vascular access device (TIVAD) to facilitate long-term venous access for frequent or continuous administration of ERT; the patient and their families should be made aware of the benefits and risks of using such a device [43, 44]. Patients with acute febrile or respiratory illness may be at increased risk of life-threatening complications from hypersensitivity reactions; therefore, the clinical status of each patient should be evaluated prior to the administration of elosulfase alfa and if necessary, delay of treatment should be considered.

***Considerations for monitoring response*** Baseline and follow-up assessments to measure treatment efficacy should be performed before, and regularly after, initiation of elosulfase alfa. These should include uGAG/KS levels, endurance testing (preferably 6MWT), respiratory function (if age compatible), growth, height and weight, pain, ADL and QoL. Thorough examination of upper and lower limb function (including active and passive range of movement and nerve conduction studies) should be performed to assess for evidence of cervical cord compression. Assessment of medical history should also be conducted to obtain as much information as possible. Stop criteria should be considered on an individual basis where the burden of infusion outweighs the benefits of treatment; however, at present this point is not universally predictable. There is currently no published evidence reporting the effects of discontinuation of ERT in MPS IVA, and additional research is required.

***Considerations for managing specific adverse events of interest*** Due to the potential for hypersensitivity reactions with elosulfase alfa, patients treated in the clinical trial programme received antihistamine premedication, with or without antipyretics, 30 to 60 min prior to the start of the infusion. This approach is broadly followed in clinical practice but there is limited evidence for or against the necessity of premedication. Patients should be closely observed for signs of anaphylaxis during and after administration of elosulfase alfa and if severe hypersensitivity reaction occurs, hospital admission is advised. Patients with a with previous history of IARs, can be given additional premedication, such as H2 receptor blockers or montelukast sodium. Other risk factors may also include history of severe allergic diathesis, status asthmaticus, reactions to other infusion or biologic products, compromised airway or pulmonary function and previous history of a significant pause between ERT treatment.

IARs are generally managed by reducing the rate of administration or by temporarily interrupting the infusion plus administration of additional antihistamines, antipyretics, or for more severe reactions, corticosteroids (for a 12–18-h period prior to infusion). Due to the risk of sleep apnoea in patients with MPS IVA, use of a non-sedating antihistamine is recommended.

##### HSCT in patients with MPS IVA (Table 5)

**Table 5:** Guidance statement for HSCT

Statement	Percentage consensus
Due to the lack of evidence, HSCT cannot be recommended for patients with MPS IVA and at this time is considered an investigational procedure Evidence Grade: D (level 3/4 studies with inconsistent risk/benefit results)	91%

***Rationale and evidence base*** Evidence supporting the use of HSCT in patients with MPS IVA is limited and comprises a small number of case studies and a single institution evaluation of ADL following surgical intervention [40, 62–66]. Evidence of GALNS expression was confirmed following transplantation, which showed the potential for cross-correction of other tissues [40, 62–66]. Improvements in height and skeletal dysplasia have not been observed but this may be due to the transplantation being performed in older patients [40, 62–66].

Due to increasing availability of well-matched donors, improved supportive care and modification to transplant regimens, the risks associated with transplantation have declined in recent years; however, mortality rates still vary between centres depending on experience, and serious risks, including death, remain. A recent study suggests that peri-transplantation mortality has improved; however, this data was from two of the most experienced centres with MPS transplantation and involved other subtypes of MPS [67].

Due to the lack of evidence related specifically to MPS IVA, and the recognised risks of transplantation, HSCT cannot be considered as a recommended therapy in patients with MPS IVA. The strongest data for the use of HSCT are for other types of MPS, namely MPS IH (Hurler syndrome) [68]. Patients with MPS IH are treated with HSCT due to the effectiveness of this approach in treating the central nervous system (CNS) manifestations of the disease, which are not improved by ERT [69–73]. In MPS IH, the increased risk of HSCT compared with ERT is considered justified. Given that CNS disease is not prominent in patients with MPS IVA, the risk–benefit profile for the use of HSCT in these patients is less clear. There are reports that the incidence of hydrocephalus and cervical stenosis is decreased in MPS IH patients treated with HSCT as compared to treatment with ERT [74]. As there are no inherent cognitive manifestations in patients with MPS IVA similar to those seen in patients with MPS IH, it is unclear whether HSCT may be advantageous in patients with MPS IVA and caution should be taken when extrapolating evidence from other MPS types [11, 17, 75]. There is a need for more research to better understand the long-term efficacy and safety of HSCT in patients with MPS IVA, and for a well-designed comparative study of HSCT and ERT in patients of similar age and disease severity.

#### Interventions to support respiratory and sleep disorders

##### Continuous positive airway pressure (CPAP), non-invasive positive pressure ventilation (NIPPV), oxygen supplementation and hypercapnia monitoring (Table 6)

**Table 6:** Guidance statements for CPAP, NIPPV, oxygen supplementation and hypercapnia monitoring

Statement	Percentage consensus
CPAP therapy is recommended for patients with MPS IVA who display the presence of obstructive sleep apnoea (OSA) that persists after tonsillectomy and/or adenoidectomy Evidence Grade: D (limited published evidence)	97%
NIPPV therapy is recommended for patients with MPS IVA who display nocturnal hypoventilation and are unresponsive to CPAP, or display daytime hypoventilation with increased PaCO_2_ and/or serum HCO_3_ levels Evidence Grade: D (level 5 expert clinical opinion)	91%
Oxygen supplementation during sleep is recommended for patients with MPS IVA who exhibit sleep apnoea with nocturnal hypoxemia, and who do not tolerate CPAP or NIPPV masks Evidence Grade: D (level 5 expert clinical opinion)	77%
Patients with MPS IVA should be monitored for development of hypercapnia after starting oxygen therapy using measurement of PaCO_2_ and/or serum HCO_3_ Evidence Grade: D (level 5 expert clinical opinion)	97%

***Rationale and evidence base*** Respiratory complications are a major cause of morbidity and mortality in patients with MPS IVA and are often among the first symptoms to appear [18, 19, 76]. Typical features of MPS IVA include upper and lower airway obstruction and restrictive pulmonary disease which result from a variety of anatomical and functional abnormalities. Upper airway obstruction is attributable to cranial abnormalities, a short neck and progressive deposition of GAGs in the tissues surrounding the supraglottic upper respiratory tract, while lower airway obstruction reflects GAG deposition in the airway walls with resultant tracheal and bronchomalacia. Lung volume and chest expansion are further limited by short stature, chest wall deformities and abdominal organomegaly. Clinical manifestations include recurrent upper and lower respiratory tract infections, obstructive sleep apnoea (OSA) and impaired exercise tolerance [18]. If respiratory complications are not diagnosed and treated appropriately, respiratory failure can ensue, leading to early death [14, 77]. There is evidence that long-term ERT is associated with sustained improvements in respiratory function in patients with MPS IVA [34]. In contrast, specific interventions beyond ERT are required to address OSA and other forms of sleep disordered breathing (SDB).

CPAP prevents upper airway collapse during inspiration and is the mainstay of treatment for OSA in the general population with benefical effects on blood pressure, cardiac events, mortality and QoL. [78, 79] A comprehensive review of the evaluation and treatment options for SDB in MPS concludes that applying CPAP can prevent functional airway collapse during inspiration when asleep and may improve obstruction-related pulmonary hypertension, heart failure and/or respiratory failure [80, 81].

An alternate form of therapy is required for patients that demonstrate either persistent OSA despite CPAP or hypoventilation during sleep. NIPPV provides an increased pressure during the inspiratory phase of breathing to augment ventilation. Although outcome data in MPS are scarce, the effectiveness of this therapy has been demonstrated in a broad range of diseases including neuromuscular and chest wall disorders; which are features that are frequently noted in patients with MPS. Evidence suggests that this approach results in an improvement in QoL, functional capacity and respiratory failure [81–83].

Supplemental oxygen can be prescribed for individuals that demonstrate persistent nocturnal oxygen desaturation and for patients who do not tolerate therapy with CPAP or NIPPV. Caution is required when prescribing oxygen because of the known suppression of respiratory drive and arousal from sleep with the potential for either worsening pre-existing hypercapnia or inducing onset of hypercapnia in susceptible patients.

***Considerations for intervention*** SDB can be managed by application of CPAP, which delivers air at an elevated pressure via a mask that fits around the nose and/or mouth; however, consideration should be given to facial abnormalities that can make mask-fitting difficult. Patients should be monitored to ensure they do not develop sustained hypoventilation. To prevent pneumonia, vaccinations against respiratory pathogens causing influenza and pneumococcus infections should be recommended.

#### Anaesthesia and surgical interventions

##### Use of anaesthesia in patients with MPS IVA (Table 7)

**Table 7:** Guidance statements for anaesthesia

Statement	Percentage consensus
Pre-, intra- and post-operative care (until extubation is complete) for all procedures requiring general anaesthesia, conscious or deep sedation, should be supervised by an anaesthetist with experience in treating patients with MPS and/or complex airway management. In addition, the anaesthetist should have access to Intensive Care support and be surrounded by an experienced team capable of performing emergency tracheotomy if required Evidence Grade: C (consistent level 4 studies)	98%
A full assessment of the risks and benefits should take place with the patient and family prior to any procedure. All pre-operative information should be made available to allow decision making Evidence Grade: C (consistent level 4 studies)	100%
ENT respiratory, cardiac, and radiological assessment should be performed prior to any procedure requiring anaesthesia Evidence Grade: C (consistent level 4 studies)	93%
It is critical to maintain a neutral neck position during all surgeries, and during intubation and extubation to avoid paralysis^a^. Strongly recommend the use of techniques that allow maintenance of the neutral neck position, including use of laryngeal mask airway (LMA) for shorter procedures, or intubation with a video laryngoscope or fibreoptic intubation Evidence Grade: C (consistent level 4 studies)	87%
Pre-operative and intra-operative measures to avoid hypotension should be adopted during all surgical procedures in patients with MPS IVA to maintain spinal cord perfusion and therefore protect spinal cord function Evidence Grade: D (limited published evidence)	98%
Intra-operative neurophysiological monitoring (including somatosensory evoked potential [SSEP], electromyography [EMG] and motor evoked potentials [MEP]) is strongly recommended during all spinal surgeries and other potentially lengthy or complicated procedures, including those that require manipulation of the head and neck Evidence Grade: D (limited published evidence)	94%
For other surgeries and procedures, neurophysiologic monitoring should be considered based on pre-existing risk for spinal cord compression and instability, need for spine manipulation, possibility of hemodynamic changes and blood loss, or extended length of time Evidence Grade: D (limited published evidence)	94%
Intrathecal and epidural techniques are high-risk in patients with MPS IVA and should be avoided wherever possible Evidence Grade: D (limited published evidence)	83%

Post-consensus comments by the SC to be taken into consideration:

^a^It is critical to maintain a neutral neck position to minimise the risk of any spinal cord injury

***Rationale and evidence base*** Patients with MPS IVA will likely require anaesthesia for multiple surgical interventions and investigations during the management of their disease [84, 85] but are considered high-risk due to potential difficulties with mask ventilation and endotracheal intubation. Other risk factors include: the presence of narrow airways from adeno-tonsillar hypertrophy and deformity of the lower airways, odontoid peg hypoplasia causing potential instability of the cervical spine, and other skeletal abnormalities causing thoracic deformity and pulmonary disposition. In addition, cardiovascular and neurological impairment may also be present [86–88].

Adverse events (including fatalities and paralysis) occurring during anaesthesia have been reported in the literature [89–91]. Intubation and extubation can be challenging in patients with MPS IVA due to several factors including: restricted mouth opening; short neck length; limited range of motion (ROM) and upper airway obstruction, which is often caused by hypertrophied tonsillar/adenoidal tissue, a large tongue with micrognathia, subglottic narrowing, and atlanto-axial instability due to odontoid hypoplasia and ligamentous laxity [77, 86, 89, 92–97]. Some patients also experience tracheal obstruction, which in the presence of coexisting upper airway obstruction commonly remains unrecognised and can increase the risk of death during anaesthesia [89, 96]. Retrospective evaluation from 83 intubations of 108 anaesthetics (in 28 patients) demonstrated difficulties in intubation following cervical fusion [89]. Airway abnormalities including tortuous appearance of the trachea and bronchi as a result of the abnormalities in the hyaline cartilage and deposits of glycosaminoglycans were observed, suggesting that MPS IVA results in abnormalities of both upper and large airways [89].

Management of the airway will require care to maintain neutrality of the cervical spine and may necessitate the use of videolaryngoscopy or fibreoptic techniques. Although hypothetical, poor perfusion related to arterial narrowing and reduced foramina diameters secondary to dysostosis should be anticipated by an anaesthetist, appropriately monitored with arterial lines, and supported in near-normal range during procedures.

Although epidural anaesthesia has been performed successfully in patients with MPS IVA [89], it is not currently recommended due to observations of spinal cord infarction following lower extremity surgeries in which patients received an epidural for post-operative pain management [91, 98, 99]. Although data on peripheral nerve block are lacking, this approach may be considered during the care of patients with MPS IVA. The use of ultrasound technology can also aid successful nerve block. Intraoperative neurophysiological monitoring is recommended to prevent significant complications in this high-risk population; however, availability is extremely variable.

***Considerations for use of anaesthesia*** Due to the risk of upper airway obstruction, pre-operative sedative premedication may be used with caution and only with appropriate monitoring. Assessment of upper and lower airway anatomy (for example, a pre-operative flexible nasopharyngolaryngoscopy and three-dimensional CT scan where feasible), cardiac function, and potential cervical spine instability should be performed prior to any procedure that requires sedation or anaesthesia. MRI scans of the spine in a neutral position or patient-initiated flexion/extension X-ray of the spine can be performed to assess the risk of spinal cord compression and instability (flexion extension X-ray measures instability only). Flexion/extension imaging is important to evaluate for cervical spine instability prior to anaesthesia. The frequency of imaging depends on both the patient’s age and clinical condition. Owing to potential unstable cervical and thoracic spinal regions, it is critical to maintain a neutral neck position during all surgeries (including intubation and extubation) to avoid spinal cord injury (which can lead to paralysis), sensory injury with dysesthesia pain and/or loss of proprioception. The anaesthetist should use techniques that allow a neutral neck position to be maintained, including use of a laryngeal mask airway for shorter procedures, or intubation with a video laryngoscope or fibreoptic scope. Use of a seated position [99] can be considered and there should be a range of options available to secure the airway and support ventilation. If possible, in a very difficult airway scenario, intubation may be completed while patients are awake, and if the patient is anaesthetised, the use of paralytic agents should be avoided such that spontaneous breathing is maintained until intubation is completed successfully. Displacing the tongue anteriorly prior to intubation by manual retraction using a ring forceps or a piece of gauze may help to access the larynx in children with MPS IVA [89]. A smaller endotracheal tube should be available and is usually necessary to avoid intraoperative swelling of the airway and enable successful extubation. Where possible, patients should be extubated in the operating room and asked to demonstrate movement of the lower extremities. If safe intubation cannot be achieved, tracheostomy may be considered electively prior to prolonged surgery, or to facilitate post-operative care. Mean arterial pressure should be maintained to maximise perfusion of the spinal cord and reduce the risk of spinal cord injury. Intensive care management is often not required but may be necessary for complicated or prolonged procedures requiring post-operative ventilation or peri-operative tracheostomy. If ventilation is performed through an endotracheal tube, it is best to aim for early extubation to minimise swelling of the airways. When clinically indicated, maintenance of intubation overnight following the procedure can be considered to allow resolution of airway swelling. Extubation should be performed by an experienced anaesthetist capable of inspecting the airway before extubation and, if necessary, performing reintubation. Wherever possible, alternative techniques (e.g. peripheral nerve block under light sedation) should be considered to avoid general anaesthesia and associated risks. However, the surgical team should always be prepared to perform general anaesthesia if required.

***Considerations after surgery*** To reduce airway oedema, intraoperative prophylaxis with steroids is the standard treatment. The use of post-operative treatment steroid prophylaxis may be required in some patients for 24 h following surgery. Standard treatment for patients with upper airway obstruction should be available including NIPPV, CPAP and continuous monitoring of respiratory and cardiac function. Intensive care management is not mandatory for all patients, but when necessary, should be maintained for 24–48 h post-surgery because of the potential complications of oral secretions, thoracic cage stiffness, heart and lung failure, apnoea, laryngospasm, bronchospasm, cyanosis and respiratory failure.

##### Limb surgeries in patients with MPS IVA (Table 8)

**Table 8:** Guidance statements for hip reconstruction, hip replacement and growth modulation surgeries

Statement	Percentage consensus
Hip reconstruction can be considered in paediatric patients with MPS IVA who exhibit hip pain, reduced walking and endurance related to hip disease, as well as abnormal radiographic findings Evidence Grade: D (limited published evidence)	86%
Hip replacement can be considered in adult patients with MPS IVA who exhibit hip pain, reduced walking and endurance related to hip disease, as well as abnormal radiographic findings Evidence Grade: D (limited published evidence)	100%
Growth modulation surgery is recommended for all patients with MPS IVA who have evidence of genu valgum and should be performed as early as possible during the period of growth Evidence Grade: D (limited published evidence)	77%

***Rationale and evidence base*** Patients with MPS IVA have progressive musculoskeletal involvement; therefore, multiple orthopaedic interventions are usually required to prevent deformity, improve physical function and reduce pain [100, 101]. Typical features of MPS IVA that do not occur in other types of MPS include joint hypermobility and deformity in the wrists, which lead to floppy wrists with weak grip and loss of fine motor skills. Patients with MPS IVA have subluxation of the hip joints and joint instability in the knees which can exacerbate genu valgum, patella dislocation and gait abnormalities [14–16, 102]. Almost all patients with MPS IVA develop genu valgum to an extent which is serious enough to require surgery [101, 103]. A review of the literature for outcomes of orthopaedic surgery suggest that correction of genu valgum by hemi-epiphysiodesis may improve QoL and function including improved walking distance (as measured by 6MWT); however, patients remain significantly impaired when compared to healthy individuals [104, 105]. A case report of knee replacements in two patients with MPS IVA also suggests that correction of genu valgum improves mechanical axis, mobility, ADL and QoL [106]. While there is more evidence available for correction of genu valgum, data for other limb surgeries are limited [104]. Evidence from case reports supports the use of hip reconstruction as early as possible (before the age of 10 years) to minimise progression of subluxation and hip dysplasia and to improve outcomes, reduce pain and facilitate hip replacement following surgical intervention. The expert SC commented that improved function has been observed following hip surgery; however, the majority of published literature suggest that the outcomes of hip surgery are judged largely on radiographic appearance with little correlation with function. Assessment of pelvic radiographs showed no correlation between 6MWT distance and degree of hip migration, and patients with hip migration greater than 40% had no increased probability of being wheelchair-bound [105]. Overall, the expert opinion of the SC group is that patients are more mobile following hip surgery; however, the literature is sparse and further data are required to support these observations.

***Patient selection for intervention*** Before orthopaedic intervention in patients with MPS IVA, morbidity and mortality risks, pain level, optimal timing, and patient preference should be considered on a case-by-case basis. The need for hip surgery can be determined by presence of hip pain, reduced walking endurance and abnormal radiographic findings indicating hip dysplasia or lower limb alignment. Growth modulation surgery should be initiated as soon as the deformity is observed, or if the tibial-femoral angle is greater than 15 degrees. For optimal results, it should be performed early during the period of growth due to the deceleration in growth that occurs as the skeleton matures, [100] however expert clinical opinion varies regarding the ideal age to perform the surgery. The period following the commencements of ERT may also be a good time to perform growth modulation surgery. Hemi-epiphysiodesis is indicated within the first decade of life, after this point osteotomy should be considered.

Currently there is no hand surgical intervention that can be recommended to improve the weakness of the grip but maintain vital flexibility for transfer and adequate ADL. External custom-made splints can be worn to help with certain tasks e.g. heavy lifting. Occupational therapists are vital to help with ADL including providing gadgets to perform necessary tasks. Patients with weak grip can learn to adapt to master necessary ADL.

***Considerations for pre- and post-surgical monitoring and assessment*** The primary goal of limb surgery is not to improve or restore joint ROM, but to reduce pain or improve mobility. Goniometer measurement performed by a physiotherapist/occupational therapist/rheumatologist may be useful, but this may not be available in all centres. Post-surgery physical assessments should be performed regularly, as patients with MPS IVA may require repeated surgeries/interventions. Patients who have undergone hemi-epiphysiodesis around the knee at one level (tibial or femoral only) and who show evidence of progression of genu valgum during follow-up should be considered for a second growth modulation procedure on the unoperated level (tibial or femoral).

***Considerations for surgery*** All surgeries should be supervised by an anaesthetist with experience in treating MPS and/or complex airway management (refer to the anaesthetics recommendations). Limb surgeries should be performed by an orthopaedic surgeon with a basic understanding of MPS including: clinical presentation, musculoskeletal abnormalities and radiographic findings. An overnight hospital stay is recommended following hip surgery to allow access to intensive care, should this be needed, although this may not be necessary for more minor surgeries, such as hemi-epiphysiodesis. Long-term intensive physical therapy is recommended post-surgery to enhance recovery.

##### Spinal surgeries in patients with MPS IVA (Table 9)

**Table 9:** Guidance statements for decompression of the spinal cord, spinal stabilisation and thoracolumbar kyphoscoliosis

Statement	Percentage consensus
Decompression of the spinal cord is recommended in patients with MPS IVA who have evidence of spinal cord compression based on clinical and radiographic findings^a^ Evidence Grade: C (level 3/4 studies)	97%
Spinal stabilisation of the craniocervical junction with either cervical fusion or occipital-cervical fusion is recommended in patients with MPS IVA who have evidence of significant instability Evidence Grade: D (limited published evidence)	97%
Correction of thoracolumbar kyphoscoliosis is recommended in patients with MPS IVA who present with progressive radiographic deformity, intractable pain and neurological deterioration Evidence Grade: C (level 3/4 studies)	100%

Post-consensus comments by the SC to be taken into consideration:

^a^The SC would like to clarify that neuroimaging is a required radiologic procedure to define compression. MRI is best to image the brain and spinal cord for this indication. Decompression of the spinal cord is recommended in patients with MPS IVA who have evidence of spinal cord compression and risk of injury based on clinical and neuroimaging findings

***Rationale and evidence base*** Spinal involvement is a major cause of morbidity and mortality in patients with MPS IVA. Early diagnosis and timely treatment of spinal stenosis/instability is critical in preventing or arresting neurological deterioration and loss of function. Spinal involvement in patients with MPS IVA occurs at three locations. Cervical involvement, particularly instability and compression at C1–C2, is very common and predisposes patients to myelopathy, paralysis and sudden death [11]. Upper cervical and craniocervical pathology is commonly seen in patients with MPS IVA; dens hypoplasia in combination with ligamentous laxity can lead to atlantoaxial instability, and subsequently, to spinal canal stenosis and spinal cord compression [107]. Although craniocervical junction instability plays a major role in cervical cord pathology in patients with MPS IVA, a retrospective analysis of 28 patients suggests that decompression surgery without occipito-cervical stabilisation may yield good postoperative results [108]. Spinal cord compression can also occur at the cervicothoracic level, and is often missed [56]. Spinal cord compression due to kyphotic deformity at the thoracolumbar level is not as common but can lead to paraplegia [102, 109, 110]. Evidence from small case studies in patients with MPS indicates that thoracolumbar spine fusion was associated with good outcomes [111, 112].

ERT and HSCT are of a limited use in preventing the development of skeletal deformities in patients with MPS IVA, therefore, early surgical intervention is important to manage neurological manifestations. Spinal instability can exacerbate spinal cord compression; therefore, a combination of multiple surgeries may be required. Based on expert experience and a few case series/studies, the consensus is that short-term post-operative outcomes are generally favourable, with high fusion rates, as well as varying degrees of neurological improvement, improved clinical outcomes, and reduction in long-term morbidity [113].

***Patient selection for intervention*** Indications for surgery include cervical spinal cord compression as determined by clinical symptoms (including weakness, numbness, paraesthesia and gait difficulty) and upper motor neuron signs or radiographic and MRI findings (including plain radiographic findings suggestive of stenosis and instability and MRI findings of extradural stenosis, cord compression, myelomalacia and instability).

Consideration should be made for timing of surgery in relation to cardiac valve replacement, as the latter procedure could subsequently commit the patient to lifetime anticoagulation therapy.

***Considerations for surgery*** Spinal surgeries should be performed by a neurosurgeon and/or spinal surgeon with a basic understanding of MPS and of the clinical presentation, musculoskeletal abnormalities and radiographic findings associated with this group of disorders [99].

##### Ophthalmic surgery in patients with MPS IVA (Table 10)

**Table 10:** Guidance statement for corneal transplantation

Statement	Percentage consensus
While significant corneal clouding is rare in patients with MPS IVA, corneal transplantation can be considered for patients with significant visual loss attributed to corneal opacification	95%
Evidence Grade: D (limited published evidence)	

***Rationale and evidence base*** The most notable ophthalmic manifestations in patients with MPS IVA are pseudoexophthalmos secondary to shallow orbits, corneal clouding retinopathy and optic neuropathy [114]. Although corneal clouding in patients with MPS IVA is generally mild, the opacification tends to worsen with age, and severe clouding has been reported in some older patients [115]. Clear corneal grafts can be achieved for patients with MPS IVA who have corneal clouding; these can improve visual acuity in some patients [116]. There were no reported cases of rejection or recurrence in studies of corneal transplantation in patients with MPS IVA [116, 117]. However, it should be noted that these studies were performed in adults and rejection rates tend to be higher in children.

***Patient selection for intervention*** Corneal transplantation should only be considered once retinopathy and optic nerve abnormalities have been assessed (by electroretinography and visual evoked potentials) and excluded as a significant contributing factor to the loss of vision. The choice of surgical technique for corneal transplantation (deep anterior lamellar keratoplasty [DALK] versus penetrating keratoplasty [PK]) should be made on a case-by-case basis. There is some evidence extrapolated from the general population to suggest that rejection is more likely to occur following PK than DALK [115, 117, 118], therefore, DALK should be considered in patients with MPS IVA.

***Considerations for intervention (e.g. if surgery)*** Monitored anaesthesia with appropriate sedation, including use of nasal CPAP/NIPPV, may be used when performing eye surgery in patients with MPS IVA. Long-term topical treatment is required following corneal transplantation, as is regular, long-term (annual) ophthalmic assessment to determine the health of the corneal graft and to check for recurrence of corneal deposits and astigmatism control. Signs of rejection require prompt ophthalmic assessment to prevent graft failure. Follow-up is required to monitor for optic neuropathy arising due to raised intracranial pressure. Symptoms can include reduction in visual acuity, abnormal pupil reactions, new onset of visual field defects or optic nerve swelling (or, more commonly optic atrophy).

##### Cardio-thoracic surgery in patients with MPS IVA (Table 11)

**Table 11:** Guidance statement for cardiac valve replacement

Statement	Percentage consensus
Cardiac (aortic, mitral) valve replacement should be considered in patients with MPS IVA who display symptomatic and severe valve stenosis or regurgitation^a^ Evidence Grade: C (level 4 studies)	95%

Post-consensus comments by the SC to be taken into consideration:

^a^The Ross procedure should not be employed in patients with MPS IVA [57]

***Rationale and evidence base*** Cardiac involvement is most commonly reported in patients with MPS I, II and VI [119]. However, results of an observational study in 54 adolescent and young adult patients with MPS IVA identified age-related aortic root dilation, thickened left-sided cardiac valves, increased heart rate and impaired diastolic filling pattern [20]. Valve dysfunction was present in 5/54 patients, with aortic regurgitation being the most common [20].

***Patient selection for intervention*** Performance and interpretation of echocardiography should be completed by physician’s familiar with expected pathological findings in patients with MPS IVA [119]. Valve replacement decisions should be based on current European Society of Cardiology (ESC), European Association for Cardio-Thoratic Surgery (EACTS) and American Heart Association (AHA) guidelines [120, 121] in conjunction with assessment of existing co-morbidities, operative risk and rehabilitation potential. Trans-catheter aortic valve replacement may be feasible for some MPS IVA patients. The Ross procedure is contraindicated in patients with systemic valvular disease [122] and should not be employed in patients with MPS IVA. Small valve annulus may preclude valve replacement with currently existing mechanical and bio-prosthetic cardiac valves. Aortic root replacement has not been reported in patients with MPS IVA.

***Consideration for surgery*** Cardiac surgery in patients with MPS IVA should be performed in a centre of excellence with a team experienced in treating both MPS and high-risk valve replacement. When possible, an anaesthetic specialist should assist the cardiac anaesthesia team during pre-operative assessment to formulate the MPS-related anaesthesia care. Airway care, including the need for tracheostomy, should be assessed on a case-by-case basis. The anaesthesia principles of care (outlined in the anaesthetics recommendations) should be followed during the care of patients with MPS IVA for cardiac procedures.

##### Ear, nose and throat surgery in patients with MPS IVA (Table 12)

**Table 12:** Guidance statements for tonsillectomy and/or adenoidectomy, tracheostomy and insertion of ventilation tubes

Statement	Percentage consensus
Tonsillectomy and/or adenoidectomy is recommended for patients with MPS IVA who display recurrent otitis media, snoring and/or OSA as early as possible following diagnosis without waiting for disease progression Evidence Grade: C (level 2, 3 and 4 studies)	94%
Insertion of ventilation tubes is recommended for patients with MPS IVA with otitis media with effusion and/or recurrent otitis media to maintain hearing and/or prevent recurrent acute otitis media Evidence Grade: D (limited published evidence)	100%
Tracheostomy is recommended in patients with MPS IVA who do not respond to any of the treatment modalities mentioned above Evidence Grade: D (limited published evidence)	77%

***Rationale and evidence base*** ENT manifestations are common in patients with MPS IVA. These often involve hearing disorders, serous otitis media and deformities of the ossicles, which can have a significant impact on patient functional status and QoL [123]. Mixed hearing loss is more common than either conductive or sensorineural hearing loss alone [124]. Evidence from two non-randomised follow-up studies indicates that ENT surgery (e.g. adenotonsillectomy, adenoidectomy with insertion of middle ear ventilation tubes, tonsillectomy, tracheotomy and exeresis of vocal cord polyps) reduces hypoacusia, otitis media, upper respiratory tract infections, occurrence of OSA, and the need for Type B tympanograms. QoL was also reported to be improved in some patients [123, 125]. The results of other non-randomised follow-up studies show that tonsillectomy and/or adenoidectomy improves OSA in the majority of MPS patients, however, the recurrence rate after adenoidectomy is high [125]. Risks include development of secondary haemorrhage following tonsillectomy and/or adenoidectomy. In addition, difficult intubations are common in these patients and can be fatal [125, 126]. Advanced surgical options such as uvulopalatopharyngoplasty, mandibular advancement surgery or tongue reduction are currently experimental. While one member of the SC had experience, there are currently insufficient data and experience to make recommendations about the use of these invasive procedures in patients with MPS IVA.

***Considerations for surgery*** Vaccinations against respiratory pathogens causing influenza and pneumococcus infections are recommended to prevent pneumonia. Insertion of ventilation tubes should be performed according to guidelines for the general paediatric population [127]. Before performing tonsillectomy, children with MPS IVA should be referred by the clinician for polysomnography to evaluate for SDB [128]. Patients who have had tonsillectomy and/or adenotonsillectomy should be observed as in-patients and may need to remain in hospital preferably in intensive care in the early post-operative period to monitor airway patency. They may need to remain hospitalised for additional days to allow close monitoring for possible haemorrhage and other complications. Patients with ventilation tubes should be assessed every 3 months, and post-operative audiologic examination should be performed if there is no improvement in hearing. The anaesthesia plan should be discussed jointly between the otolaryngologist and anaesthesia care team. Precautions to prevent spinal cord compression should be taken during surgical procedures.

## Discussion

MPS IVA and VI are rare, heterogeneous and progressive disorders that are associated with severe morbidity and reduced mortality. Although previously published guidance is available for the management of MPS IVA/VI [11, 17, 40, 75, 129, 130], these were largely based on clinical opinion among a relatively small group of experts and therefore the methodology used to generate this guidance has come under increased scrutiny. This programme used a robust, systematic approach to develop consensus-based guidance among a broad group of experts to improve the holistic care of patients with MPS IVA/VI (the results for MPS VI can be found in the companion article (*Recommendations for the management of MPS VI: systematic evidence- and consensus-based guidance*).

Developing guidance in rare diseases, such as MPS IVA/VI, is often challenging. The low patient numbers often mean that there is a paucity of large-scale randomised trials and systematic reviews available in the literature. As such there is a need for robust consensus-based guidance to bridge the gap between the relatively small number of clinicians with experience in treating patients with MPS IVA/VI, and the broader community of healthcare professionals involved in the management of these patients. However, with consideration of the challenges including lack of evidence and limited resources, guidelines remain critical for driving consistency and quality of care in rare diseases [131].

### Strengths and limitations of the programme

Management of MPS IVA/VI requires a coordinated multidisciplinary approach; therefore, it was important that this guidance covered a broad range of topics. However, as is common with rare diseases, high-quality evidence was not available for all the medical and surgical interventions covered in this guidance. As a result, it was necessary to draw on the expert clinical experience of the SC and broader modified-Delphi respondents to develop consensus-led guidance. Key strengths of this programme included the robust methodology used to identify and coordinate experts from a wide range of specialties and geographic spread, the systematic review of available literature and consensus on a broad range of guidance statements. This was achieved through a systematic approach to select the programme Co-Chairs and identify appropriately-qualified respondents for the modified-Delphi voting. Additionally, the inclusion of three PAG representatives as part of the SC group and interview analysis from six global PAGs, ensured that the patient perspective was reflected in the guidance. The comprehensive systematic literature review ensured that the resulting guidance was developed on the current evidence base and the use of a blinded modified-Delphi voting process to gain consensus ensured that the guidance reflected the views of a wide range of specialists with specific experience in managing the complex needs of patients with MPS IVA/VI. The methodological rigour and transparency employed within the development of the guidance was further demonstrated through the review of the manuscripts against the validated AGREE II instrument (www.agreetrust.org). Three independent reviewers assessed the manuscripts and suggested amendments were addressed where possible; a subsequent second round of review was conducted by all reviewers. The manuscripts were given an overall guideline assessment score of 5.3/7 (where 1 represents the lowest quality, and 7 represents the highest quality).

Limitations of the programme reflect other challenges of developing guidance in rare diseases including achieving multi-sponsor funding. While this programme was sponsored by a single funder, measures were taken to ensure this did not influence the final guidance statements (outlined in the methods and process section). Due to the limited funding available within the field of rare diseases, additional sponsorship for this programme was unavailable. However, the Steering Committee group encourages future multi-sponsored consensus programmes for updates to this guidance and in other MPS types/rare diseases.

Access to relatively expensive therapies such as ERT, as well as access to other medical and surgical care can be challenging for exceptionally rare diseases. Patients’ access to treatment in rare diseases varies geographically based upon national reimbursement schemes. It is important that inclusion/exclusion criteria for treatment is not arbitrary and does not discriminate against slowly progressing patients, where a health gain cannot often be confirmed over a short period of time [35, 132]. Consideration should also be given to the unique challenges of rare diseases, which are often underserviced by investment, with relatively high costs, which may not be captured by conventional cost-benefit analyses. Given that price and health systems vary across the world, and these guidelines are intended for global use, it was difficult to apply one cost analysis to these guidelines, therefore consideration of cost analyses was not included in this process.

### Facilitators and barriers to the implementation of the guidance

The PAG consultations provided patient and caregiver insight into the barriers and facilitators to the application of this guidance across numerous healthcare systems and geographies, and the results were reviewed and further considered from a clinical perspective by the SC group. It was considered that patients and their families are likely to drive the dissemination and application of this guidance among healthcare professionals and that this can be supported by making the guidance widely available, for example, via the websites of MPS societies. To address knowledge barriers for healthcare professionals, the SC recommends that a number of materials should be developed including: case studies to support implementation of the guidance statements (e.g. using local experience that demonstrates the benefit of early diagnosis and treatment); a guidance document to provide advice on specific questions that should be asked and data that should be collected at each clinic visit; and guidance and training on how to develop an emergency plan for patients as it is not always possible to get an MDT together quickly. The SC also recommended the development of a frequently asked questions (FAQ) document for use by PAGs and patient organisations. Following publication of the guidance, members from the SC group will partner with organisations such as PAGs and pharmaceutical companies to further understand the facilitators and barriers to the application of the guidance, including cost considerations. Subsequent support programmes will then be developed to deliver the recommended actions within the guidance.

The SC highlighted the benefits that existing industry-sponsored MPS registries play in supporting data collection and discussed how these may provide important data on outcomes of medical and surgical interventions. One example is the elosulfase alfa NICE-managed access agreement in England, which aimed to collect data on the long-term efficacy and safety of elosulfase alfa as well as prevalence and effectiveness of other treatment paradigms [133]. The SC group discussed the value of the continuation of such registries and suggested that these could be run by a range of stakeholders beyond industry. The SC recommends that patients should be empowered to take an active role in their care with emphasis being placed on the increasing importance of their role in generating data (e.g. patient-reported outcome measurements [PROMs]). The development of international, regional multidisciplinary centres could be used to optimise monitoring, treatment and data collection. Further, improved recording of data (particularly those relating to HSCT) through participation in international registries will help to address evidence gaps, assist treatment decisions and improve outcomes for patients with MPS IVA/VI.

### Future directions

Improvements in symptom management and the introduction of ERT for MPS IVA/VI may create new challenges for the future. As life expectancy increases, the challenge will be to manage a growing population of adult patients who may require multiple surgeries throughout their lifetime and therefore the associated anaesthetic risks will gain greater importance. Similarly, the introduction of newborn screening will facilitate early treatment and has the potential to change the natural history of MPS IVA/VI. MPS I has already been added by the Secretary of the Department of Health and Human Services to the list of routine recommended conditions to be screened in the USA, and the inclusion of other MPS types should follow as testing technology becomes more established and the benefits of early diagnosis and treatment become clearer. Biochemical enzyme assays continue to be important for differential diagnosis of MPS types and several assays for enzymes relevant to MPS IVA/VI have been described [134–138].

A novel urine assay has been developed that allows diagnosis of 10 subtypes of MPS in a single test by measuring oligosaccharides with non-reducing termini in urine samples to provide a diagnostic oligosaccharide signature unique for each MPS subtype. These disease-specific oligosaccharides can be used to monitor response to therapeutic interventions [48]. A fluorometric assay for *N*-acetylgalactosamine-6-sulfate (GALNS) has been developed for the differential diagnosis of MPS IVA; however, the data suggest this will not be robust enough for newborn screening [139]. Ongoing pilot studies of more accurate tandem mass spectrometry assays and their application to newborn screening have been described [140]. Furthermore, advances to improve the accuracy of newborn screening for LSDs by reducing the number of false positive results are already underway [141].

Evidence suggests that although most patients develop anti-drug antibodies to elosulfase alfa, immunogenicity does not appear to impact efficacy or correlate with the frequency of IARs [142, 143]. However, further studies are required to fully understand the clinical implications of anti-drug antibody (ADA) responses in MPS. A case report in MPS II suggests that urinary GAG levels, measured by 1,9-dimethylmethylene blue (DMB) assay, may be a biomarker for anti-idursulfase neutralising antibodies [144]. This study highlights the importance of regular and frequent monitoring of urinary GAG levels in patients with MPS II who are receiving ERT. The SC highlighted the need for further investigation of biomarkers and assays in MPS IVA/VI with timely publication of findings to inform guidance on monitoring. It was suggested that when improved assays become available, consideration should be given to measurement of total and neutralising antibodies every 6 months in patients receiving ERT, with a rapid turnaround of laboratory results.

Future therapies, including gene therapy, small molecule therapy, and other anti-inflammatory agents such as pentosan polysulphate (PPS) [145] are being investigated for the treatment of MPS, and trials are ongoing. The small molecule, odiparcil, restored a normal corneal structure in the eye of a genetic mouse model of MPS VI [146]. Given that ERT has a limited impact on bone abnormalities and current therapies do not relieve skeletal symptoms, it will be important to assess the effect of new therapies (such as gene therapy) on skeletal manifestations to address this unmet need. Tumour necrosis factor alpha (TNF-α) inhibition has been suggested as a potential adjunctive therapy to help manage pain in individuals receiving ERT and/or HSCT based on the observation that increased levels of TNF-α are associated with pain and decreased physical function [147].

Across all therapies, an individualised dosing approach would address one of the main challenges for the pharmacological treatment of patients with MPS. Treatment would be greatly enhanced with the definition of minimal clinically important differences for MPS and the development of improved tools and/or biomarkers for monitoring response to therapy. The development of MPS-specific patient-reported outcome measures is also needed, along with formal standardisation and validation of health-related QoL tools for the different MPS subtypes [148].

## Conclusions

This manuscript provides robust evidence- and consensus-driven guidance that can be used by all healthcare professionals involved in the management of patients with MPS IVA; results for MPS VI are detailed in the companion article (*Recommendations for the management of MPS VI: systematic evidence- and consensusbased guidance*). The guidance is intended for use by healthcare professionals that manage the holistic care of patients with MPS with the intention to improve clinical- and patient-reported outcomes and enhance patient quality of life. It recognised that the guidance provided represents a point in time and further research is required to address current knowledge and evidence gaps. The SC recommends that this guidance is reviewed and updated within 5 years, or sooner if there are significant changes to medical practice.

## Additional files

Additional file 1: Methodology: Further information regarding methodology, including: defining clinical questions using the P.I.C.O methodology, the search strategy recording form, results of the systematic literature review according to PRISMA, the Oxford Centre for Evidence-based Medicine criteria and the AGREE II evaluation. (DOCX 69 kb)

Additional file 2: Oxford CEBM grading for MPS IVA**:** Tables detailing the evidence levels given to each reference supporting the MPS IVA guidance statements and the Evidence Grades applied to each guidance statement. Evidence levels were assessed using the Oxford Centre for Evidence-based Medicine and were based on the quality of evidence of each reference. For each guidance statement, an overall Evidence Grade was applied, based on the evidence levels of the supporting references. (DOCX 41 kb)

Additional file 3: Oxford CEBM grading for MPS VI**:** Tables detailing the evidence levels given to each reference supporting the MPS VI guidance statements and the Evidence Grades applied to each guidance statement. Evidence levels were assessed using the Oxford Centre for Evidence-based Medicine and were based on the quality of evidence of each reference. For each guidance statement, an overall Evidence Grade was applied, based on the evidence levels of the supporting references. (DOCX 41 kb)

Additional file 4: Modified-Delphi voting Round 1**:** Full results of the first round of the modified-Delphi voting, which was used to demonstrate consensus of the guidance statements. (DOCX 152 KB) (DOCX 151 kb)

Additional file 5: Modified-Delphi voting Round 2**:** Full results of the second round of the modified-Delphi voting, which was used to demonstrate consensus of the guidance statements. (DOCX 41 kb)
